# *PsANT*, the adenine nucleotide translocase of *Puccinia striiformis*, promotes cell death and fungal growth

**DOI:** 10.1038/srep11241

**Published:** 2015-06-10

**Authors:** Chunlei Tang, Jinping Wei, Qingmei Han, Rui Liu, Xiaoyuan Duan, Yanping Fu, Xueling Huang, Xiaojie Wang, Zhensheng Kang

**Affiliations:** 1 State Key Laboratory of Crop Stress Biology for Arid Areas and College of Plant Protection, Northwest A&F University, Yangling, China; 2 State Key Laboratory of Crop Stress Biology for Arid Areas and College of Life Science, Northwest A&F University, Yangling, China

## Abstract

Adenine nucleotide translocase (ANT) is a constitutive mitochondrial component that is involved in ADP/ATP exchange and mitochondrion-mediated apoptosis in yeast and mammals. However, little is known about the function of ANT in pathogenic fungi. In this study, we identified an ANT gene of *Puccinia striiformis* f. sp. *tritici* (*Pst*), designated *PsANT*. The *PsANT* protein contains three typical conserved mitochondrion-carrier-protein (mito-carr) domains and shares more than 70% identity with its orthologs from other fungi, suggesting that ANT is conserved in fungi. Immuno-cytochemical localization confirmed the mitochondrial localization of *PsANT* in normal *Pst* hyphal cells or collapsed cells. Over-expression of *PsANT* indicated that *PsANT* promotes cell death in tobacco, wheat and fission yeast cells. Further study showed that the three mito-carr domains are all needed to induce cell death. qRT-PCR analyses revealed an in-planta induced expression of *PsANT* during infection. Knockdown of *PsANT* using a host-induced gene silencing system (HIGS) attenuated the growth and development of virulent *Pst* at the early infection stage but not enough to alter its pathogenicity. These results provide new insight into the function of *PsANT* in fungal cell death and growth and might be useful in the search for and design of novel disease control strategies.

Apoptosis is a form of cell death that plays key roles in development, tissue homeostasis and disease^1^. The misregulation of apoptosis might lead to degenerative diseases, such as the Alzheimer disease in humans^2^. Apoptosis is governed in cells by a sophisticated machinery with an elaborate array of checks and balances^3^. Mitochondria contribute to apoptosis induction by changing mitochondrial membrane permeability (MMP), and an *in vitro* system for apoptosis induction requires the presence of mitochondria^4^. The lethal change in MMP results from a primary mitochondrial outer membrane permeabilization (MOMP) and the mitochondrial permeability transition (MPT) in the mitochondrial inner membrane^5^. MPT activation compromises the normal integrity of the mitochondrial inner membrane, which makes the inner membrane freely permeable and allows the free redistribution of solutes and water across the inner membrane. This finally results in matrix expansion and mechanical rupture of the outer membrane^6^. MPT is regulated by the opening of the permeability transition pore complex (PTPC), a supramolecular complex that is assembled at the contact sites between the mitochondrial outer and inner membranes through the dynamic interaction of multiple proteins, including voltage-dependent anion channels (VADC), adenine nucleotide translocase (ANT) and cyclophilin D^7^. ANT is a constitutive mitochondrial inner membrane ADP-ATP antiporter that imports ADP into the mitochondria and exports ATP to the cytoso. The ANT dimer exists in two conformations that are referred to as the matrix (m) and cytosolic (c) states, in which ADP-ATP is bound to either the matrix side or the cytosolic side of the inner membrane, respectively. The MPT activity is determined by the orientation of the nucleotide site of ANT. Thus, carboxyatractyloside favors the c-state of ANT-induced MPT, whereas bongkrekate favors the m-state of ANT-inhibited MPT^8^.

The role of ANT in the regulation of cell death by MPT is conserved across nematodes and mammals. In *Caenorhabditis elegans*, there are four isoforms of worm ANT (WAN-1, 2, 3 and 4), but only the knockout of WAN-1 yields a lethal phenotype. Shen *et al.*^9^ reported that WAN-1 forms a complex with CED-4 (the nematode counterpart of Apaf-1) and CED-9 (a Bcl-2 ortholog). The BH3-only protein EGL-1 disrupts the interaction between CED-9 and WAN-1, which leads to osmotic swelling of the mitochondrial matrix and eventually to the permeabilization of mitochondrial outer membrane. Four closely related isoforms of ANT (ANT1, 2, 3 and 4) exist in humans, and ANT1 and ANT3 are pro-apoptotic proteins^10^,^11^, whereas ANT2 exerts pro-survival functions^12^. The ability of ANT4 to modulate apoptosis has not yet been investigated. The main processes and key players that drive programmed cell death and apoptosis in mammalian cells display a high level of conservation throughout evolution. However, some important differences exist, such as the release of cytochrome c (Cyt c) from the mitochondrial intermembrane space, an event that seems to occur exclusively in mammalian cells^5^. Meanwhile, genetic evidence has suggested that ANT makes a phylogenetically old contribution to the cell death machinery in *Saccharomyces cerevisiae*. The absence of all three ANT isoforms (aac1, aac2 and aac3) in yeast protects cells exposed to acetic acid and diamide from cell death^13^.

Functional analyses of genes in yeasts revealed that the progression of apoptotic –like programmed cell death (PCD) in the single-celled fungus yeast resembles that of higher eukaryotes at the molecular level^14^,^15^. Apoptotic cell death also occurs in the model pathogenic fungi *Candida albicans*^16^,^17^, *Aspergillus fumigates*^18^,^19^ and *Magnaporthe oryzea*^20^. The discovery of the apoptosis response makes it possible to control fungal diseases by developing novel antifungal drugs and fungicides that could activate fungal cell apoptosis. However, in contrast to the increasing number of studies that are beginning to unravel the apoptosis pathway in yeasts, comparatively little work has focused on pathogenic fungi. Comparative analysis of the genome sequence of four *Aspergillus* and seven other fungal species revealed more than 100 apoptosis-associated genes, including lineage-specific proteins as well as a conserved core component of the ancestral apoptosis machinery that is shared by all fungi, suggesting that there is a complex uncharacterized regulatory network in fungi^21^. In addition, homologs of several mammalian apoptotic proteins, including PARP and AMID, have been found in filamentous fungi^21^, indicating that the molecular pathways that control apoptosis in different organisms are conserved even though they differ in complexity. Furthermore, the overexpression of Cyclophilin D *PaCypD* accelerated organism aging and cell death induction in *Podospora anserina*^22^. The deletion of *PaAif2* and *PaAmid2*, two genes encoding mitochondrial apoptosis-inducing factors (AIFs) such as oxidoreductases, led to increased stress tolerance and lifespan extension^23^. In addition, bioinformatic screening of fungal genomes also revealed that many of the known components of apoptosis in higher eukaryotes are missing or show high divergence, indicating that pathogenic fungi may possess a distinctive apoptosis pathway.

Identification of the molecular switches that trigger fungal apoptosis is of paramount importance in generating drug therapies. However, despite the existence and cell-death-regulating function of CYPD and AIF in fungi, little is known about the cell death machinery in pathogenic fungi. In this study, we obtained an ANT homolog *PsANT* in the pathogenic rust fungi *Puccinia striiformis* f. sp *tritici* (*Pst*) which devastates wheat crops worldwide^24^. The *PsANT* protein contains three typical mito-carrier domains and is located in the mitochondria of infected *Pst* hyphae. *PsANT* exhibited a pro-cell-death effect in fission yeast, wheat and tobacco cells. Furthermore, knockdown of *PsANT* in *Pst* using host-induced gene silencing (HIGS) blocked the growth and development of *Pst* in the early infection stage but did not alter the virulence of *Pst*.

## Results

### Analyses of the deduced *PsANT* protein

**Figure 1:**
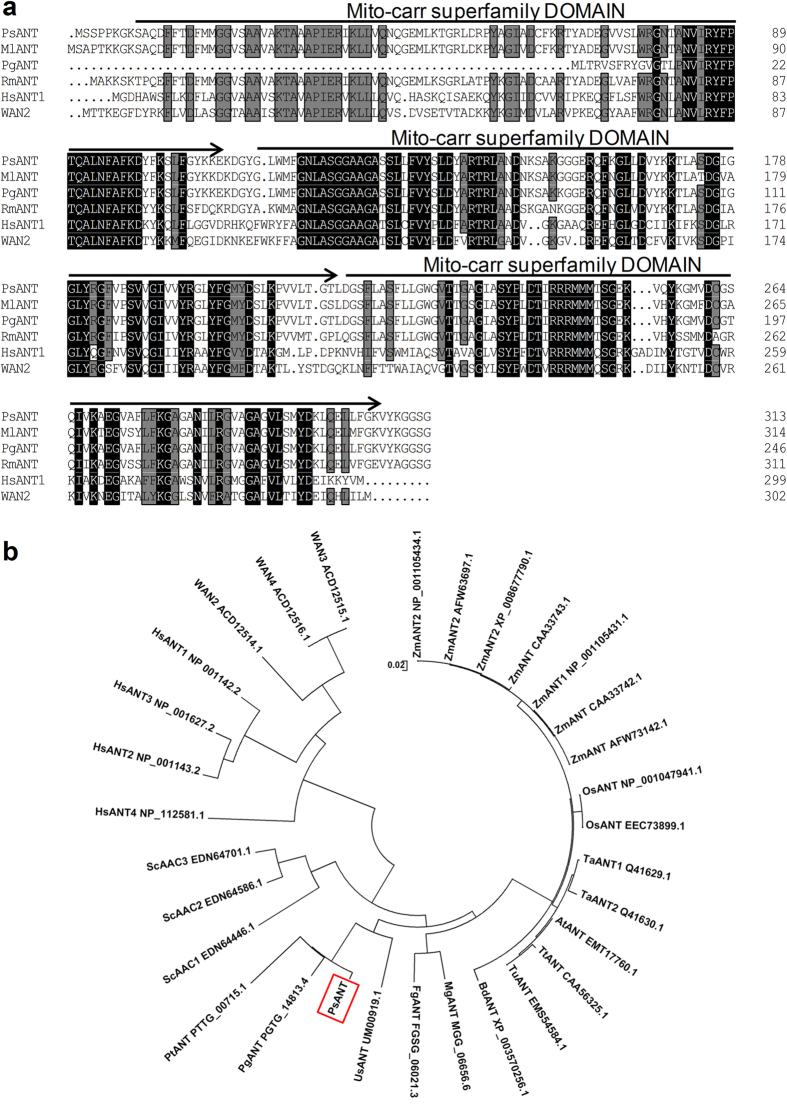
Multi-sequence alignment and phylogenetic analysis of *PsANT* and other members of the ANT family in other species. (**a**) Protein multiple alignment. Identical and similar amino acid residues are shaded in black and light gray, respectively. Lines with arrows indicate the three conserved mito-carr super-families. (**b**) Phylogenic analysis of *PsANT* and other ANT family members using MEGA 4.1 software. Branches are labeled with protein names and GenBank accession numbers. Ps: *Puccinia striiformis* f. sp*. tritici*; Pg: *Puccinia graminis* f. sp*. tritici*; Pt: *Puccinia triticina*; MI: *Melampsora larici-populina*; Rm: *Rhodotorula mucilaginosa*; Us: *Ustilago maydis*; Mg: *Magnaporthe grisea*; Fg: *Fusarium graminearum*; Sc: *Saccharomyces cerevisiae*; and Hm: *Homo species*; Tt: *Triticum turgidum*; Tu: *Triticum urartu*; At: *Aegilops tauschii*; Ta: *Triticum aestivum*; Zm: *Zea mays*; Os: *Oryza sativa*; Bd: *Brachypodium distachyon*.

An 1182-bp full-length *PsANT* cDNA sequence was obtained with an open reading frame of 945 bp. The deduced *PsANT* protein consisted of 313 amino acids and showed the highest similarity with MlANT (93.9%) in *Melampsora larici-populina* (*Mlp*). A multi-sequence alignment with various ANT sequences in the National Center of Biotechnology Information database and Broad Institute database revealed that *PsANT* shared more than 70% identity with its orthologs from other fungi, including *Mlp*, *Puccinia graminis* f. sp. *tritici* (*Pgt*) and *Saccharomyces cerevisiae* (Fig. 1a). In contrast, *PsANT* had only 51.68% identity with HsANT1 in humans and 46.4% to WAN2 in *C*. *elegans*. The primary structure of *PsANT* contained three conserved 100-amino-acid-long mito-carr domains (Fig. 1a).

A phylogenetic analysis categorized *PsANT* and its homologs into two distinct groups (Fig. 1b). ANT homologs in fungi, including the single-celled yeast fungus and different multi-cellular pathogenic fungi, were clustered into one large clade, while the homologues in humans and *C. elegans* were grouped into another clade. Interestingly, the ANT homologues from plants were found to exist in the clade characterized by the fungi members, indicating a closer relationship of ANT in fungi and plants.

### *PsANT* protein localized in mitochondria of the invasion hyphae of *Pst*

**Figure 2:**
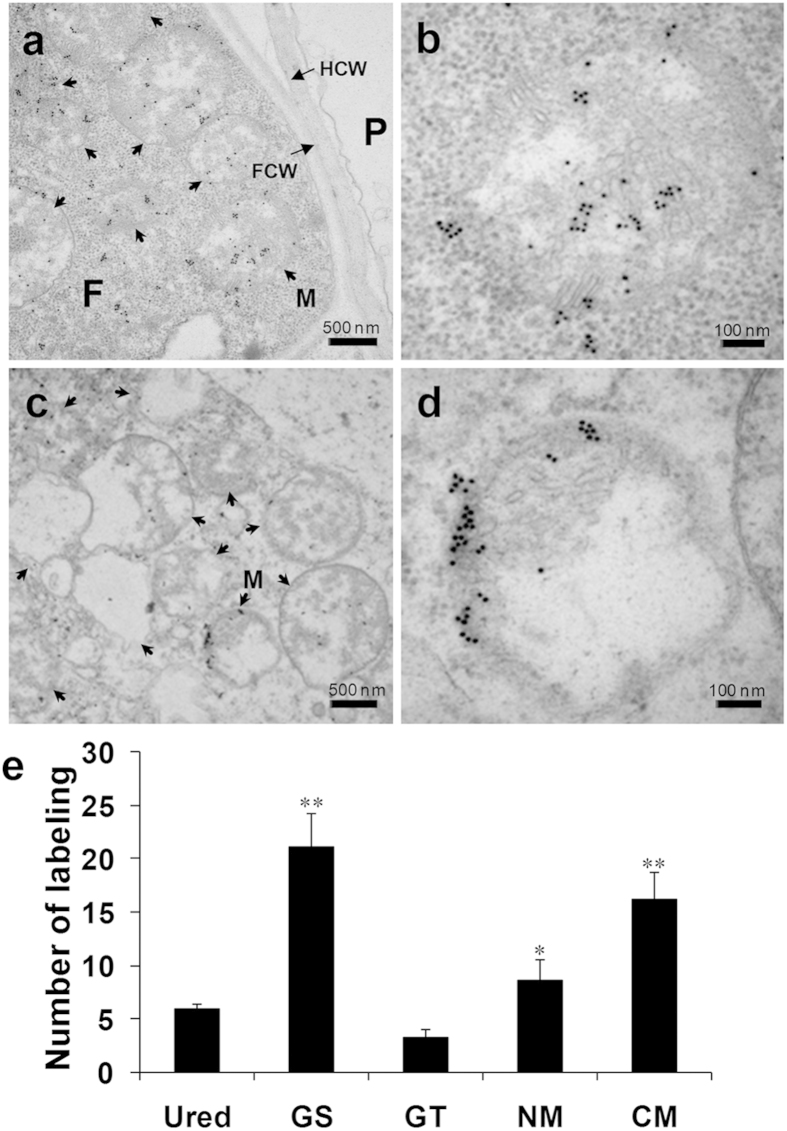
Distribution of *PsANT* in the mitochondria of invading hyphae of *Puccinia striiformis* f. sp*. tritici* by immuno-cytochemical localization. Electron micrographs of rust-infected wheat leaves 5 d after inoculation. Sections were probed with anti-*PsANT* antiserum and detected with goat anti-rabbit secondary antibodies that were conjugated to 10-nm gold particles. (**a,b**) shows the mitochondrial location of *PsANT* in the healthy fungal infection hyphal cell. Labeling occurs in the mitochondria. (**c,d**) shows the mitochondrial distribution of *PsANT* in hyphal cells undergoing collapse. (**e**) Comparison of ANT amounts in each mitochondria before and after *Pst* invasion in wheat using immuno-cytochemical localization. The results were obtained from calculation of *PsANT* labeling from 50 mitochondria. And three biological replications were performed. Data are presented as mean ± SE. Asterisks indicate a significant difference (*P < 0.05, **P < 0.01) verus uredinospores using Student’s *t* test. Ured: uredinospore; GS, germinated uredinospores; GT, germ tube; NM, mitochondria under normal condition; CM, collapsed mitochondria;. P, plant; F, fungus; M, mitochondria; FCW, fungal cell wall; and HCW, host cell wall.

To obtain more information about *PsANT* in the pathogenic fungi *Pst*, immuno-cytochemical localization was used to characterize the functional site of *PsANT* in *Pst*. Labeling with the primary antibody prepared from the 61 amino acids (40–100) of *PsANT*. *PsANT* was localized to the mitochondria of the infectious hyphae during the compatible interaction of wheat and *Pst* (Fig. 2), confirming that *PsANT* is a member of mitochondrial protein. The tight texture of the cells and the regular shape of the mitochondria indicated the active physiological status of the invasion hyphal cells, and the concentration of *PsANT* in the mitochondria (Fig. 2a,b) may indicate its involvement in energy supply during invasion and hyphal development. Interestingly, *PsANT* labeling was also found in mitochondria in collapsed cells, in which the cytoplasm was broken down and the mitochondria were aberrant (Fig. 2c,d).

Furthermore, to address the role of *PsANT* during infection of *Pst*, we compared the amounts of *PsANT* in uredinospores, germinated uredinospores, germ tubes and invasion hyphae. The results showed that before invasion, much more abundant labeling was observed in germinated uredinospores than in uredinospores and germ tubes (Fig. 2e), indicating that the energy for germination were mainly supplied by uredinospores and *PsANT* was responsible for the energy supply due to its translocase activity. After invasion, in the invasion hyphae, more labeling of *PsANT* was found in mitochondria under normal condition (NM) compared to uredinospres, but less than in germinated uredinospores. This may due to that during germination, the energy supply mainly rely *PsANT*, and after invasion, *Pst* also could uptake energy through haustoria from host plants besides depending on *PsANT*. Interestingly, more *PsANT* labeling was observed in collapsed mitochondria (CM) than in mitochondria under normal condition. This may indicate that except for energy supply role, *PsANT* also participate in mitochondria mediated apoptosis.

### Over-expression of *PsANT* leads to cell death in fission yeast with accumulated reactive oxygen species (ROS)

**Figure 3:**
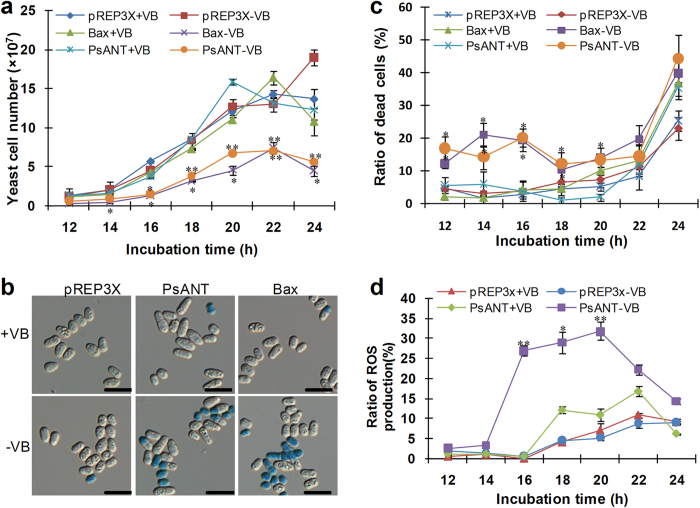
Over-expression of *PsANT* induced cell death in fission yeast cells. (**a**) Fission yeast cells carrying pREP3X, pREP3X_*Bax* or pREP3X_*PsANT* were incubated in yeast medium in the absence (−VB) or presence of (+VB) 5 μg/mL thiamine. pREP3X and pREP3X_*Bax* were used as the negative and positive control, respectively. With an identical initial OD_600_ at 0.01, the number of yeast cells per milliliter was calculated at 12, 14, 16, 18, 20, 22 and 24 h. Overexpression of *PsANT* under the repressed promoter of pREP3X without thiamine (*PsANT*-VB) triggered significant cell death with much less fission yeast cells compared to that with thiamine (*PsANT*+VB). The fission yeast cells were counted using a hemocytometer and the values were obtained from 3 replicates per treatment and three biological replicates were performed. (**b**) The cell death phenotype of yeast cells were checked by methylene blue staining. The dead yeast cells were stained into blue. Bars = 10 μm. (**c**) The ratio of dead yeast cells in total cells was calculated. Stained cells were counted in ten view fields for each treatment. Fission yeast cells expressing *PsANT* or *Bax* resulted in more cell death than the controls. (**d**) The H_2_O_2_ level in fission yeast cells transformed with pREP3x and pREP3x_*PsANT* was determined by DHR123 staining. Stained cells were counted in ten view fields under a fluorescence microscope for each treatment. The proportion of yeast cells with H_2_O_2_ was measured. Fission yeast cells expressing pREP3X_*PsANT* resulted in more abundant H_2_O_2_ accumulation than the controls. Values were collected from 10 view fields per treatment and three biological replicates were performed. Data are presented as mean ± SE. Asterisks indicate a significant difference (*P < 0.05, **P < 0.01) from treatment with thiamine using Student’s *t* test.

Because of the lack of an available transformation system in *Pst*, we studied the role of *PsANT* in cell death by the aid of fission yeast system. *PsANT* was expressed in yeast driven by the nmt promoter, which is repressed by thiamine. *Bax* and the pREP3x empty vector were used as the positive and negative controls, respectively. Expression of *Bax* resulted in obvious cell death from 12 h to 16 h, whereas pREP3x did not change fission yeast growth (Fig. 3a). Fission yeast cells expressing *PsANT* in absence of thiamine (−VB) led to a significant reduced fission yeast cell number compared to those in presence of thiamine (+VB) from 16 h to 24 h after incubation (Fig. 3a).

To determine whether expression of *PsANT* or *Bax* leads to cell death or reduced yeast growth, we checked the cell death phenotype of treated yeast cells with methylene blue staining. As shown in Fig. 3b, through methylene blue staining, the dead yeast cells were stained into blue. Furthermore, we calculated the ration of stained yeast cells in total cells. The results showed that yeast cells expressing *PsANT* incubated without thiamine (−VB) exhibited significantly higher level of dead cells than that incubated with thiamine (+VB) from 12 to 20 h (Fig. 3c). Expression of *Bax* in yeast cells resulted in significant cell death as observed for *PsANT* (Fig. 3c). The fission yeast cells transformed with empty pREP3x did not show much change in ratio of dead cells. Thus, the results suggested that *PsANT* induce cell death in fission yeast cells, and the reduced number of fission yeast expressing *PsANT* or *Bax* was due to cell death but not reduced growth.

DHR123 staining of H_2_O_2_ in yeast cells revealed that from 16 h to 24 h, the oxidant level in *PsANT* over-expressing yeast cells without thiamine was significantly higher than in those that were incubated with thiamine (Fig. 3d). The fission yeast cells transformed with empty pREP3x did not show much change in ROS accumulation. Thus, the results suggest that the cell death induced by *PsANT* in fission yeast cells is characterized by accumulation of H_2_O_2_.

### Over-expression of *PsANT* leads to cell death in wheat

**Figure 4:**
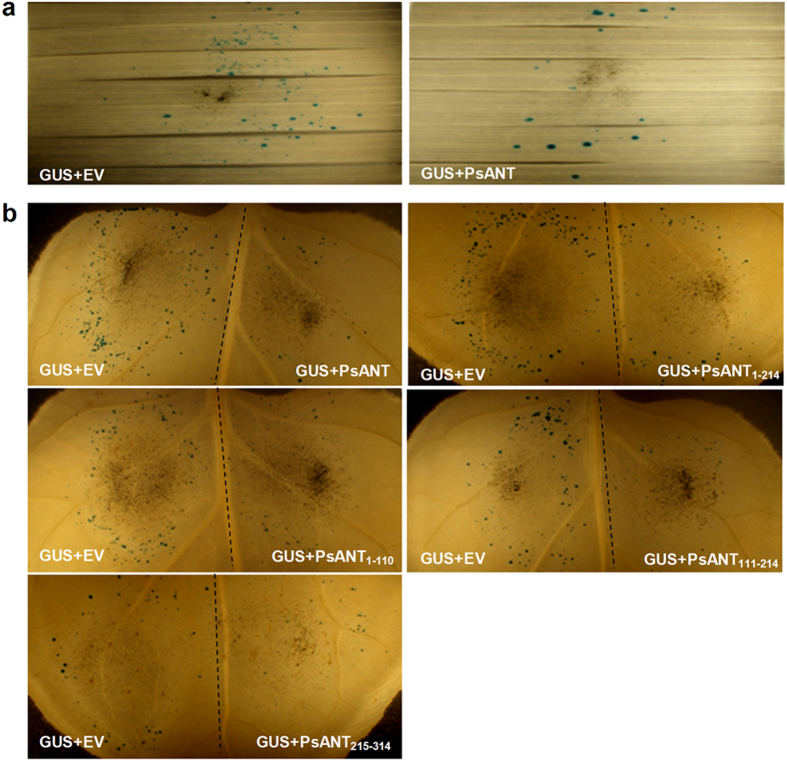
Over-expression of *PsANT* through particle bombardment triggered cell death in *N. benthamiana* and *T. aestivum* leaves. (**a**) Over-expression of *PsANT* in *T. aestivum* leaves using a single-barreled particle bombardment. The DNA mixtures used to bombard different groups of leaves are indicated. (**b**) Transient expression of *PsANT* and truncated mutants of *PsANT* in *N. benthamiana* leaves using double-barreled particle bombardment. The leaves were bombarded with the indicated pair of DNA mixtures. The dotted line indicates the position of a divider that was used to prevent overlap of the two bombardment areas. Statistical analysis of results from 14 shots was conducted for each assay. EV, empty vector.

Given the close relationgship of ANT in fungi and plants, we addressed whether *PsANT* could induce cell death in the host of *Pst*. The single-barreled particle delivery system was used to deliver *PsANT* and *GUS* into wheat leaves. The β-glucuronidase (*GUS*) gene was introduced into tobacco leaf cells as a reporter, and the contribution of the protein encoded by *PsANT* to cell death was measured by the restoration of cells expressing *GUS*. When the wheat leaves were bombarded with *PsANT* + *GUS*, a 64.2% reduction in the number of GUS blue spots was observed compared to leaves that were shot with empty vector plus *GUS* (*EV* + *GUS*) (Fig. 4a).

### The three mito-carr domains contributed differently to cell death induction

As a member of the mitochondria carrier family, *PsANT* consists of three 100-residue mito-carr repeats. To determine the domains that are responsible for cell death induction, a double-barrel various deletion mutants of *PsANT* were tested in tobacco leaves, which is easier to operate as a model plant. A particle bombardment assay with a double-barreled attachment was applied to facilitate the direct comparison of test and control bombardments. Expression of *PsANT* reduced the number of GUS-positive blue patches by 78.7% compared to controls, verifying the cell death induction role of *PsANT* (Fig. 4b and Table 1). Then, truncated *PsANT* mutants were further examined. Transient expression of PsANT_1–110_, PsANT_111–214_ or PsANT_215–314_, which contained the first, second or last mito-carr domain, resulted in a 45.9%, 54.7% and 40% reduction of the blue spots, respectively. Meanwhile, transient expression of PsANT_1–214_, which comprised the first two mito-carr domains, led to a decrease of approximately 70% in GUS spots compared to the control (Fig. 4b and Table 1). The results indicated that the three mito-carrier domains contribute differently to cell death and likely work together to engage in cell death induction.

### *PsANT* was up-regulated during infection of *Pst* in wheat

**Figure 5:**
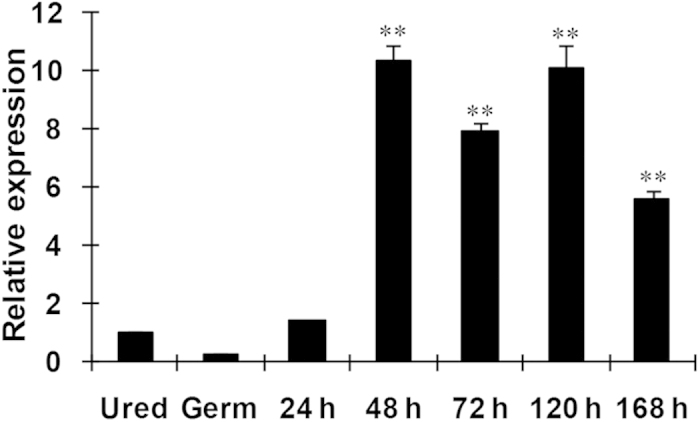
Expression of *PsANT* during the different development stages of *Puccinia striiformis* f. sp. *tritici*. Three independent biological replications were performed. The expression levels were normalized to *PsEF-1a*. Three biological replicates for each time point were averaged with standard error of mean indicated. Asterisks indicate a significant difference (**P < 0.01) from urediniospores using Student’s *t* test. Ured, uredinospores; Germ, germ tubes.

qRT-PCR analyses revealed that *PsANT* transcripts were detected at relatively low levels in urediniospores and germ tubes but were sharply induced at 48 hpi and kept constant until 5 dpi (Fig. 5). The results indicate an in-planta induction of *PsANT* in the infection and propagation stage of the stripe rust fungus.

### Silencing of *PsANT* attenuated *Pst* growth and development

**Figure 6:**
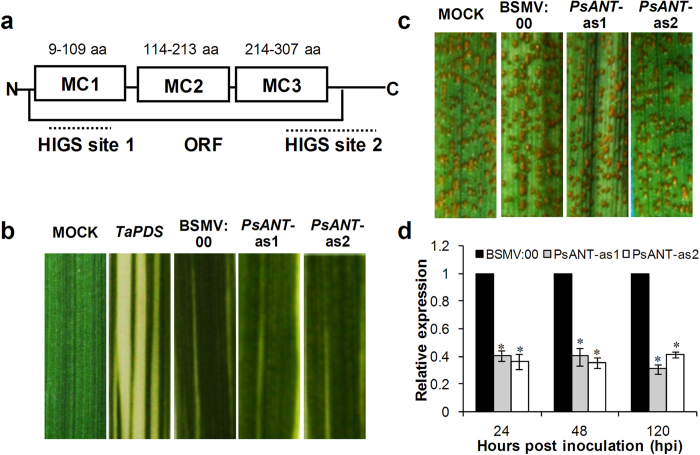
Functional analyses of *PsANT* during the interaction between wheat and stripe rust using the BSMV-mediated host-induced gene silencing system (HIGS). (**a**) The two fragments that were used to silence *PsANT*. (**b**) Mild chlorotic mosaic symptoms were observed on the 4^th^ leaves of the seedlings at 9 dpi with BSMV, and photobleaching was evident on the 4^th^ leaves of plants that were infected with BSMV:*TaPDS*. MOCK: Wheat leaves with Fes buffer. (**c)** Disease phenotypes of the 4^th^ leaves that were pre-inoculated with BSMV:00, BSMV:*PsANT*-as1 or BSMV:*PsANT*-as2 and then challenged by virulent CYR31. (**d**) Silencing efficiency assessment of *PsANT* in the *Pst*. The 4^th^ leaves inoculated with BSMV:00 followed by inoculation with CYR31 were used as the controls. The data were normalized to the *PsEF-1a* expression level. Three biological replicates for each time point were averaged with standard error of mean indicated. Asterisks indicate a significant difference (*P < 0.05) from BSMV:00 using Student’s *t* test.

Due to the lack of a stable genetic transformation system for stripe rust fungi, the host-induced gene silencing (HIGS) technique mediated by BSMV (Barley stripe mosaic virus) was used to knockdown the expression of *PsANT* in *Pst*. Two fragments were designed for silencing as indicated in Fig. 6a. Considering the existence of ANT in wheat, we obtained the ANT in wheat variety Shuiyuan 11 (Su11) (Su11-ANT) and compared *PsANT* with *Su11-ANT* to examine the specificity of the silencing fragment. *PsANT* shared a similarity of 65.84% with *Su11-ANT* at the nucleotide level. No more than 11 consecutive identical nucleotides were observed between the targeted *PsANT* and *Su11-ANT* (Fig. S1). This should prevent off-target effects while maintaining adequate complementarity for target gene silencing^25^.

Nine days after inoculation with BSMV, obvious photo bleaching was observed in *TaPDS*-knockdown plants (Fig. 6b), suggesting that the BSMV-HIGS system worked well. The rust disease phenotypes photographed at 14 dpi showed that *Pst* with decreased *PsANT* was still virulent enough to cause a full susceptible phenotype of wheat with no significant change compared to the control plants (Fig. 6c).

qRT-PCR analyses showed that the transcript level of *PsANT* was knocked down by approximately 69% in the leaves that were infected with BSMV:*PsANT* compared to that in the BSMV:00-infected leaves (Fig. 6d). The results indicated that *PsANT* was partially knocked down and confirms that silencing of *PsANT* did not alter the pathogenicity of *Pst*.

### Silencing of *PsANT* impeded the growth and development of *Pst*

**Figure 7:**
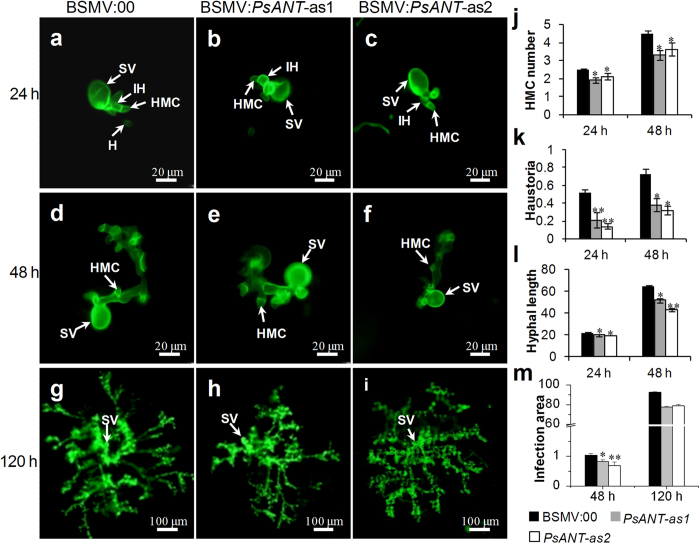
Histological observation of fungal growth in whea*t* challenged by virulent *Puccinia striiformis* when *PsANT* was knocked down. Wheat leaves that were pre-infected with BSMV:00 or recombinant BSMV were followed by inoculation with *Pst* CYR31 in the compatible interaction. The fungal structure was stained with wheat germ agglutinin (WGA). The fungal growth of *Pst* pathotype CYR31 in wheat leaves that were inoculated with BSMV:00, BSMV:*PsANT*-as1 and BSMV:*PsANT*-as1 at 24 hpi (**a–c**), 48 hpi (**d–f**) and 120 hpi (**g–i**) was observed under a fluorescence microscope. Average number of haustorial mother cells (HMC) (**j**) and haustoria (**k**) of *Pst* in each infection site were counted. (**l**) Hyphal length, which is the average distance from the junction of the substomatal vesicle and the hypha to the tip of the hypha, was measured by DP-BSW software (unit in μm). (**m**) Infection area, the average area of the expanding hypha plus the host cells, was calculated by DP-BSW software (unit in × 10^3^ μm^2^). All the results were obtained from 50 infection sites and three biological replications were performed. Asterisks indicate a significant difference (*P < 0.05, **P < 0.01) from BSMV:00 inoculated plants using a two tailed Student’s *t* test. SV, substomatal vesicle; HMC, haustorial mother cell; IH, infection hypha; H, haustoria.

Despite of the unchanged disease phenotype, the development and growth of *Pst* with silenced *PsANT* were examined through histological observation. The 4^th^ leaves were sampled at 24 and 48 hpi, when primary haustoria began to form in mesophyll cells and when hyphal growth began. As shown in Fig. 6, the silencing of *PsANT* decreased the number of haustorial mother cells and haustoria at 24 hpi and 48 hpi (Fig. 7a–f,j,k). Meanwhile, hyphal growth was also significantly reduced, with shorter hyphae at 24 hpi and 48 hpi and a smaller infection area at 48 hpi (Fig. 7a–f,l,m). However, by 120 hpi when haustoria formed in great number, the infected area of *Pst* with silenced *PsANT* was slightly but not significantly smaller than that of the controls (Fig. 7m).

## Discussion

ANT is a physiologically conserved component of programmed cell death in mammals and *C. elegans* that regulates the permeability of the mitochondrial membrane as a component of the permeability transition pore (PT)^5^. Genetic evidence also suggests that ANT makes a phylogenetic contribution to cell death in another ancient organism, yeast. However, little has been investigated regarding the role of ANT in plant pathogenic fungi. In this study, we identified an adenine nucleotide translocase homologue *PsANT* in the wheat stripe rust fungus. *PsANT* contained three typical conserved mito-carr domains of ANT members in yeast and mammals, indicating the conservation of ANT across fungi and animals. Interestingly, different from the multiple ANT members found in other organisms, only one single ANT was discovered in *Pst* in this study. Since the pathogenic fungus mainly depend on their host for nutrient and energy supply, their own energy supply mechanism might have been degenerated during the evolution, and only one ANT member might have remained to supply energy for pathogen growth before setup of the parasite relationship on host.

To investigate the function of *PsANT*, we analyzed the location of *PsANT* in *Pst* during infection in wheat. Immuno-cytological localization showed that *PsANT* was concentrated in the mitochondria of the invasion hyphae, confirming that *PsANT* was a member of the ANT family in the mitochondria. Mitochondrial labeling of *PsANT* was observed in normal hyphal cells as well as in collapsed cells. ANT is a bi-functional protein that under the normal physiological condition exerts the ATP/ADP translocation role and under apoptotic conditions can be converted into a pro-apoptotic pore under the control of onco- and anti-oncoproteins from the Bax/Bcl-2 family in mammals^26^,^27^. Thus, combined with the abundant *PsANT* in germinated uredinospores and the invasion hyphae, the localization of *PsANT* in mitochondria under the normal condition may be responsible for supplying energy for the germination of uredinospores and development of the invading *Pst* hyphae through its ATP/ADP translocase activity Whereas, the abundance of *PsANT* in collapsed mitochondria also indicates that *PsANT* is involved in apoptosis-like cell death in *Pst*. Our results provide direct evidence for the mitochondria localization of ANT members in a plant pathogenic fungus, which may coincide with their bi-functional role in energy supply and mitochondria mediated cell death.

Because the lack of a stable transformation system for use in *Pst*, heterologous system was applied to assess the temporal relationship between *PsANT* and cell death. Exploiting the yeast system, *PsANT* was shown to induce cell death as observed for mouse pro-apoptotic *Bax*. The pro-cell death role of *PsANT* was consistent with the lethal effect of the three ANT isoforms in yeast^28^,^29^. Similarly, ANT1 and ANT3 of human were reported to act as pro-apoptosis factors that can dominantly induce apoptosis when over-expressed in human cell lines^10^. Therefore, it is presumable that ANT functions as a conserved cell death component across fungi and mammals. In fact, an earlier comparative analysis of the programmed cell death pathways in filamentous fungi has revealed the existence of conserved core components of the ancestral PCD machinery in Metazoa^21^, which indicated filamentous fungi and Metazoa shared a conserved PCD machinery.

Coping with the close evolution relationship of ANT in fungi and plants, we found that transient expression of *PsANT* promoted significant cell death in the monocotyledon *Triticum aestivum* and dicotyledon *N. benthamiana*. Based on the well established double-barrel bombardment assay in tobacco leaves, we determined the domains of *PsANT* that are responsible for cell death induction in tobacco. The three mito-carr domains were all required to induce cell death, but made different level of contribution. The second domain made a greater contribution compared to the first and last one. The combination of the first two domains led to more cell death than any single domain but was still not as efficient as the complete *PsANT* protein. Therefore, we presumed that the three domains worked together to induce cell death. However, a previous study reported that the first three transmembrane helixes of ANT-1 in humans are responsible for inducing apoptosis, and the remaining two transmembrane domains are not required^10^. Considering that a truncated molecule could not form a membrane channel, it was suggested that overexpressed ANT-1 might have triggered cell death through a PT-independent mechanism. The difference between these findings in humans and our observations of *PsANT* might suggest that the mechanism by which ANT induces cell death differs between pathogenic fungi and mammals.

The function of ANT has been well studied in mammals and yeasts, but due to the limitations of the yeast system, the role of ANT in pathogen pathogenicity is unclear. The detection of *PsANT* transcripts in the entire infection stages of *Pst* suggested that *PsANT* was of fundamental function to *Pst*. The in-planta-induced expression of *PsANT* during the biotrophic stage indicated that *PsANT* is important for *Pst* infection, but there is little direct functional evidence to show which genes are involved in rust pathogenicity and virulence because mutagenesis and transformation protocols are lacking in *Pst*. Recently, host-induced gene silencing (HIGS) was developed as a novel tool to address gene function in obligate biotrophic fungi. The expression in plants of RNA interference (RNAi) constructs targeting fungal genes can specifically silence their targets in the invading pathogenic fungus *Blumeria graminis*^30^. Gene fragments from the rust fungi *Puccinia striiformis* f. sp. *tritici* and *P. graminis* f. sp. *tritici* were delivered to plant cells through the *Barley stripe mosaic virus* (BSMV) system, and some of these fragments reduced the expression of the corresponding genes in the rust fungus^31^. Thus, we studied the likely role of *PsANT* in the growth and pathogenicity of *Pst* through knocking down *PsANT* during the interaction of wheat and *Pst* using the BSMV-mediated HIGS system. *PsANT* was efficiently silenced by 40%–60%, and the partial silencing of *PsANT* led to blocked development and growth of *Pst* with attenuated formation of haustorial mother cells and haustoria at early stages of infection. Along with this reduction in haustorial formation, hyphal growth and infection were also repressed. In the early stage of infection in host plants, when haustoria have not yet formed in large numbers, *Pst* depends mainly on the energy supplied by *PsANT* for invasion and expansion. The results indicate that *PsANT* plays a role in maintaining the normal development and differentiation of *Pst* at an early stage through its energy supply function. However, despite the inhibited development at the early stage, the infected area was approximately the same as that of the controls at late stage, suggesting that the pathogenicity of *Pst* was not fundamentally affected in *PsANT*-knockdown plants. This may due to that at the late infection stage, haustoria begin to form in great numbers and the expansion of *Pst* mainly depends on the nutrient and energy uptake from host cells through haustoria. Thus, the silencing of *PsANT* did not substantially influence *Pst* infection during the late stage. These results indicate that *PsANT* contributes to fungal growth and development probably due to its energy supply role during early infection but is not essential for virulence. This possibly could further explain the lower copy numbers of ANT in *Pst* than in its host wheat and other speicies.

In conclusion, our study demonstrated that *PsANT* promotes cell death and contributes positively to the growth and development of *Pst*. These findings suggest that fungi, plants and animals shared phylogenetically conserved components in the cell death mechanism. Further studies addressing how *PsANT* modulate fungal cell death will be required to share light on the concrete cell death mechanism of pathogenic fungi. Notwithstanding that, the study of fungal cell death and pathogenicity mechanism will be of great help in designing of fungicides for controlling the disease.

## Methods

### Plant materials, strains and growth conditions

*Puccinia striiformis* f. sp. *tritici* (*Pst*) pathotype CYR31 and wheat genotype Su11 were used in this study. *Pst* race CYR31 was propagated on Su11 as described by Kang &Li^32^. Fresh urediniospores were harvested from infected wheat leaves and dispersed uniformly in sterile distilled water. After incubation at 9 °C for 10 h, germinated urediniospores were collected, quickly frozen in liquid nitrogen and stored at −80 °C for RNA extraction. To study the expression of *PsANT* during infection, wheat leaves that were infected with *Pst* CYR31 were sampled at 24, 48, 72, 120 and 168 h post-inoculation (hpi). For each assay, three biological replicates were applied.

*Nicotiana benthamiana* plants, which were used for transient over-expression by particle bombardment, were grown at an ambient temperature of 25 °C under a light cycle of 16 h light/8 h dark.

### Total RNA extraction and qRT-PCR

Total RNA of urediniospores, germ tubes and wheat leaves that were infected with *Pst* were isolated using TRIzol Reagent (Invitrogen, Carlsbad, CA, USA). A 2.0-μg RNA aliquot of each sample was used for cDNA synthesis with an Oligo(dT)_18_ primer using the Reverse Transcription PCR system (Promega, Madison, WI, USA). The resulting 1st-strand cDNA products were used as the templates for qRT-PCR. qRT-PCR was conducted as described by Wang *et al.*^33^ with a 7500 Real-Time PCR System (Applied Biosystems, Foster City, CA, USA). The *Pst* elongation factor *Pst_EF* gene^31^ was used as the internal reference for each qRT-PCR reaction. All of the reactions were performed in triplicate, and reactions without templates were used as negative controls. The comparative 2^–ΔΔCT^ method was used to quantify relative gene expression^34^. The primers for qRT-PCR are listed in Supplementary Table 1.

### Isolation of cDNA and sequence analysis

The primers *PsANT* _F and *PsANT* _R were designed on the basis of EST sequence WRIS_455 (GenBank # GR304380) from a cDNA library that was constructed from wheat that was infected with the virulent *Pst* pathotype CYR31^35^. The open reading frame (ORF) was amplified from the 1^st^-strand cDNA that was synthesized with RNA from the urediniospores of *Pst* race CYR31.

The deduced amino acid sequence of *PsANT* was analyzed with PFAM for conserved domains and motifs. TMPRED was used to predict the trans-membrane domains. Multiple sequence alignments were carried out using DNAMAN software, and phylogenetic analysis of *PsANT* and other ANT members was performed using the neighbor-joining method.

### Plasmids construction

The plasmid pUCPsANT that was used in transient expression in tobacco and wheat leaves was constructed as described by Dou *et al.*^36^ PUCPsANT (*PsANT* driven by the CaMV 35S promoter) was obtained by inserting the coding sequence of *PsANT* into *Xma* I- and *Kpn* I-digested plasmid pUCBAX (*BAX* gene driven by the CaMV 35S promoter). Truncated mutants of *PsANT*, including pUCPsANT_1–110_, pUCPsANT_111–214_ and pUCPsANT_215–314_, which contained the first, second and last mito-carrier domains, respectively, were generated in a similar manner, as was the pUCPsANT1–214 construct, which represented the first two conserved mito-carrier domains.

To generate the *PsANT* construct that was used for bacterial expression in *E. coli*, primers were designed to amplify a 186-bp fragment with a stop codon, which encodes the amino acid sequence of *PsANT* from residues 40 to 100. The 186-bp fragment was inserted into pET-32a(+) vector (Novagen) that had been digested with *BamH*I and *Hind*III.

For the overexpression assay in yeast, the coding sequence of *PsANT* was amplified. The PCR products were digested with *Xho*I and *BamH*I and inserted into the digested pREP3x vector (driven by repressed nmt promoter)^37^. pREP3x_Bax was constructed by inserting the coding sequence of Bax into *Xho*I- and *Sal*I-digested pREP3x vector.

A BSMV γRNA-based vector was constructed as described by Holzberg *et al.*^38^ The cDNA fragments derived from the coding sequence and the 3' untranslated region, which were 190 bp (949–1138) in size, and from the coding sequence, which were 202 bp (131–332) in size, were used to construct the recombinant *PsANT*-as1 and *PsANT*-as2 plasmids, respectively. To guarantee the specificity of gene silencing, the fragments that showed the highest polymorphism within the gene family and the lowest sequence similarities with other genes using a BLASTN search in the NCBI database were chosen to construct γRNA-based derivative plasmids.

The primers for all of the plasmid constructions are documented in Supplementary Table S1.

### Immuno-cytochemical localization

The prokaryotic expression of the *PsANT* protein in *E. coli* strain BL21 (DE3) with the generated construct and the preparation of the primary polyclonal antibody in rabbit were completed by Hangzhou Anhua Biotechnology Co., Ltd., China. For the immuno-cytochemical localization of *PsANT*, the samples were prepared and analyzed as previously described^39^. For immuno-electron microscopy, the primary leaves of wheat that was infected with *Pst* race CYR31 were harvested 5 dpi. Small leaf specimens (~2 mm^2^) were excised from the infected tissues and fixed with 3% glutaraldehyde (v/v) in 100 mM phosphate buffer (pH 6.8) for 3 h at 4 °C. After washing in phosphate buffer four times (15 min each), the leaf sections were post-fixed with 1% (w/v) OSO4 in 100 mM phosphate buffer (pH 6.8) and again washed four times for 15 min each. The samples were then dehydrated in a graded ethanol series and embedded in LR-white resin (Berkshire, U.K.). The embedded samples were cut with a vibratome (TPI, Series 1000) to obtain ultra-thin sections. After incubation at 9 °C for 6 h, germinated urediniospores were collected. The samples for immuno-cytochemical localization of *Pst* uredinspore and germinated uredinospores were prepared as described for the wheat leaf samples.

The sections were blocked three times for 20 min each with 1% (w/v) BSA in PBS (50 mM Na-phosphate, 150 mM NaCl, pH 7.2) and incubated with the rabbit anti-*PsANT* antibody (1:1000) in 1% BSA for 2 h at room temperature. After three washes in 1% BSA (15 min each) and two washes in PBS, the sections were incubated with the secondary colloidal gold antibody (goat anti-rabbit IgG conjugated to 15 nm gold particles (1:20) diluted with PBS for 1 h at room temperature. The samples were post-stained with 5% (w/v) uranyl acetate in 50% (v/v) ethanol for 8 min and in lead citrate for 6 min. The sections were examined with a Zeiss-EM10 transmission electron microscope (Tokyo, Japan) at an 80-kV accelerating voltage.

### Transient expression of *PsANT* in tobacco and wheat by particle bombardment

The leaves of 5-7-week-old *N. benthamiana* plants were used for the transient expression of *PsANT*. Over-expression of *PsANT* was achieved through particle bombardment assays using the Bio-Rad He/1000 particle delivery system with an attached double-barreled extension^36^. For bombardment, 100 μL of 90 mg/mL tungsten particles was combined with 50 μg of empty vector and 50 μg of GUS plasmid (pUCGUS) or a mixture of 50 μg of pUCPsANT and 50 μg of pUCGUS. The prepared tungsten-DNA mixture was used for 30 shots. To quantify the role of *PsANT* in cell death, empty vector (1.67 μg/shot) and pUCGUS (1.67 μg/shot) were bombarded onto half of the tobacco leaf in one barrel as controls. In the second barrel, the pUCPsANT plasmid (1.67 μg/shot) and pUCGUS (1.67 μg/shot) were delivered into the other half of the tobacco leaf. After incubation at 28 °C for 3 d in darkness, the bombarded leaves were stained for 16 h at 28 °C using 0.8 mg/mL X-gluc (5-bromo-4-chloro-3-indolyl-b-D-glucuronic acid, cyclohexylammonium salt); 80 mM Na phosphate, pH 7.0; 0.4 mM K_3_Fe(CN)_6_; 0.4 mM K_4_Fe(CN)_6_; 8 mM Na_2_EDTA; 0.8 mg/mL 20% methanol; and 0.06% (v/v) Triton X-100 and then de-stained in 100% ethanol. For each shot pair (GUS+*PsANT* DNA versus GUS+control DNA), the logarithm of the ratio of the blue spots on the leaves that were bombarded with GUS and *PsANT* to that of the control barrels was calculated using the Wilcoxon signed ranks test. At least 16 shot pairs were performed.

For the over-expression of *PsANT* in wheat leaves, the first leaves of 7-day-old wheat Su11 were used in the particle bombardment assay. The wheat leaves were packed tightly into dishes and shot with the Bio-Rad He/1000 single-barreled particle delivery system according to the previously described protocol^40^,^41^. All of the plasmids that were used were prepared at a concentration of 1 μg/μL. For bombardment, 26.7 μL of 90 mg/mL tungsten particles was mixed with 7 μg of the GUS plasmid (pUCGUS) and 7 μg of the empty vector or 7 μg of pUCPsANT, after which an equal volume of 1 M Ca(NO3)_2_ was added. After incubation for 10–30 min at room temperature with periodical inversion, the mixtures were centrifuged at 14,000 rpm for 30 s. Then, the pellets were successively washed with 1 mL of 70% ethanol and 1 mL of 96% ethanol. Finally, the pellet was re-suspended in 30 μL of 96% ethanol. For each shot, 4 μL of the prepared DNA-tungsten mixture was applied. The leaves were kept at 28 °C in darkness for 2 days and then stained as described above for 16 h and de-stained in 100% ethanol. The blue shots that formed on the leaves that were shot with GUS and *PsANT* plasmids were compared to those that formed on the leaves that were shot with GUS and empty vector. Each assay consisted of seven shots and was conducted at least twice. Significant differences between the treatment and controls were analyzed with a two tailed Student *t*-test.

Several truncated mutants of *PsANT* were constructed and tested in tobacco to identify the functional domain of *PsANT*. pUCPsANT1–110 and pUCPsANT_1–214_ excised the last two mito-carrier domains and the last mito-carrier domain, respectively. pUCPsANT_111–214_ and pUCPsANT_215–14_ contained the middle mito-carrier domain and the last mito-carrier domain, respectively.

### Over-expression of *PsANT* in fission yeast

The reconstructed vector pREP3x_*PsANT*, pREP3x_*Bax* and pREP3x empty vector were transformed into *Schizosaccharomyces pombe* by electroporation. Thiamine was used as the repressor of the nmt promoter in the pREP3x vector at a concentration of 5 μg/mL. Positively transformed cells were verified by PCR with pREP3x_F and pREP3x_R primers. The positively transformed cells were incubated in EMM medium with a starting optical density of OD_600_ = 0.01 for 24 h with or without thiamine. The incubated fission yeast cells were sampled at 12, 14, 16, 18, 20, 22 and 24 h post incubation, and the fission yeast cells were counted using a hemocytometer. The cell death phenotype of treated yeast cells were checked by methylene blue (Sigma-Aldrich, St. Louis, MO) staining as described by Fraser^42^. The dead yeast cells were stained into blue and the ratio of dead yeast cells in total cells were calculated from at least 10 view fields. The levels of mitochondria oxidants in fission yeast cells were determined by dihydrorhodamine 123 (DHR123) staining as previously described^43^. The stained yeast cells were observed under an Olympus BX-51 fluorescence microscope (Olympus Corp., Tokyo) and measured from at least 10 view fields. The proportion of yeast cells with ROS accumulation was calculated. For all the experiments three biological replications were performed.

### BSMV-mediated silencing of *PsANT* in the compatible wheat-*Pst* interaction

Capped transcripts were prepared from the linearized plasmids containing the tripartite BSMV (Barley stripe mosaic virus) genome using the mMESSAGE T7 *in vitro* transcription kit (Ambion Austin, TX, U.S.A.) following the manufacturer’s instructions. The second leaf of the wheat seedlings at the two-leaf stage was inoculated with BSMV transcripts by gently rubbing the surface with a gloved finger^38^,^44^. The BSMV-infected plants were maintained in a growth chamber at 23 ± 2 °C. Nine days after BSMV infection, the fourth leaves were inoculated with fresh urediniospores of the *Pst* pathotype CYR31. The disease symptoms of the fourth leaves were recorded at 14 days post-inoculation (dpi) with rust fungi. Three independent sets of wheat plants were prepared for each of the four BSMV constructs (BSMV:00, BSMV:*PsANT*-as1, BSMV:*PsANT*-as2 and BSMV:*TaPDS*) with a total of 72 seedlings. *TaPDS* encodes the *Triticum aestivum* phytoene desaturase, which has been used as control of virus-induced gene silencing^44^. Another 18 seedlings were mock inoculated with 1×Fes buffer^45^ and used as the control. Three independent biological replicates were performed.

The fourth leaves were sampled at 24, 48 and 120 hpi for qRT-PCR and histological observation. The silencing efficiency of *PsANT* was confirmed using qRT-PCR as described above.

### Histological observation of fungus growth

To determine the effects of silencing *PsANT* in stripe rust fungus, fungal development was examined with a microscope. The sampled wheat leaves were fixed and stained as previously described^46^. Cleared segments were examined with an Olympus BX-51 microscope (Olympus Corp., Tokyo) for infection sites. Only an infection site on which substomatal vesicles had formed in the stomata was considered a successful penetration. At least 50 infection sites were examined for each of the five randomly selected leaf segments for each treatment. Wheat germ agglutinin (WGA) conjugated to Alexa 488 (Invitrogen, Carlsbad, CA, USA) was used to stain the infection structure of *Pst* as previously described^47^. The hyphal length, hyphae branches and haustorial mother cells were observed and calculated using DP-BSW software. Standard deviations and Student’s *t*-test were applied for statistical analysis.

## Additional Information

**How to cite this article**: Tang, C. *et al.* *PsANT*, the adenine nucleotide translocase of *Puccinia striiformis*, promotes cell death and fungal growth. *Sci. Rep.* **5**, 11241; doi: 10.1038/srep11241 (2015).

## Supplementary Material

Supplementary Information

## Figures and Tables

**Table 1:** Induction of PCD by transient expression of *PsANT* in tobacco.

Experiment	Barrel 1^a^	Barrel 2^b^	Direct ratio^b^	*P*value^d^
A	PsANT+GUS	EV+GUS	0.21 ± 0.04	*P*<0.01
B	PsANT_1–110_ +GUS	EV+GUS	0.54 ± 0.07	*P*<0.05
C	PsANT_111–214_ +GUS	EV+GUS	0.45 ± 0.06	*P*<0.05
D	PsANT_215–314_ +GUS	EV+GUS	0.60 ± 0.07	*P*<0.05
E	PsANT_1–214_ +GUS	EV+GUS	0.33 ± 0.05	*P*<0.05

^a^Barrels 1 and 2 were physically identical, and the masses of DNA in each barrel were identical. All of the replicates were conducted on the petiole-proximal half of the leaves and then the petiole-distal half. EV, empty vector.

^b^Log ratios of the blue spots in barrel 1 versus barrel 2. The geometric averages and SE were calculated from the log ratios that were obtained from 16 pairs of shots.

^d^The *P* value for the direct comparison was calculated from the log ratios using the Wilcoxon signed-ranks test. A significant *P* value (*P *< 0.05) indicates that cell death was induced by *PsANT*.
