# Supplementary material for: PsANT, the adenine nucleotide translocase of Puccinia striiformis, promotes cell death and fungal growth
Source: Sci Rep. 2015 Jun 10;5:11241. doi: 10.1038/srep11241 (PMC4462048; doi:10.1038/srep11241)
Supplement: Supplementary Information [file srep11241-s1.pdf]

Supplementary Information

PsANT, the adenine nucleotide translocase of *Puccinia striiformis*, promotes cell death and fungal growth

Chunlei Tang[1], Jinping Wei[1], Qingmei Han[1], Rui Liu[1], Xiaoyuan Duan[2], Yanping Fu[2], Xueling Huang[1], Xiaojie Wang[1*] and Zhensheng Kang[1*]

[1]State Key Laboratory of Crop Stress Biology for Arid Areas and College of Plant Protection, Northwest A&F University, Yangling, China

[2]State Key Laboratory of Crop Stress Biology for Arid Areas and College of Life Science, Northwest A&F University, Yangling, China

*Corresponding author: Zhensheng Kang and Xiaojie Wang

State Key Laboratory of Crop Stress Biology for Arid Areas

Yangling, Shaanxi, 712100, China

Tel: 86-2987080061

Fax: 86-2987080061

Email: kangzs@nwsuaf.edu.cn; wangxiaojie@nwsuaf.edu.cn

```
                                                 Start codon
PsANT       GGTTGTCGCTTGATCTCACCCAAGTACTTCTTTTCAACATGTCGTCTCCTCCAAAGGGCAAGA.................     63
SU11-ANT    ......................................ATGTCATCTTCTCCATTGTTTGCCAATGCACCCCCTGAGAAG     42

                                                                                  HIGS site 1
PsANT       ....GTGCCCAAGATTTCTTCACCGATTTCATGATGGGAGGTGTCTCCGCCGCGGTTGCCAAGACCGCCGCAGCACCCAT    139
SU11-ANT    AAAGGTGTCAAGAACTTTGCGATTGACTTCCTCATGGGAGGAGTGTCAGCTGCAGTTTCCAAGACTGCTGCAGCTCCTAT    122

PsANT       TGAGCGTATCAAGCTCTTGGTTCAGAACCAGGGTGAGATGCTCAAGACTGGTCGTTTGGACCGACCTTACGCTGGTATTG    219
SU11-ANT    TGAGCGTGTTAAGCTGCTTATCCAGAACCAGGATGAGATGATCAAGGCTGGCAGGCTCTCTGAGCCATACAAGGGTATTG    202

PsANT       CCGATTGTTTCAAGCGTACTTACGCCGATGAGGGTGTCGTGTCTTTATGGCGTGGTAACACTGCCAACGTAATCCGATAC    299
SU11-ANT    GTGACTGCTTTGGGCGCACCATCAAGGATGAAGGCTTTGGCTCACTGTGGAGAGGAAACACTGCTAACGTCATCCGTTAC    282

PsANT       TTCCCTACCCAGGCCCTCAACTTCGCCTTCAAGGATTACTTCAAGTCCCTGTTCGGTTACAAGAAGGAGAAGGATGGTTA    379
SU11-ANT    TTCCCAACTCAGGCTTTGAACTTTGCATTCAAGGATTACTTCAAGAGGATGTTCAACTTCAAGAAGGACAAGGATGGTTA    362

PsANT       CGGACTCTGGATGTTTGGTAACTTGGCCTCTGGTGGTGCCGCTGGTGCCTCATCCCTGCTCTTTGTCTACTCGCTCGATT    459
SU11-ANT    CTGGAAGTGGTTCGGTGGCAACCTTGCTTCTGGTGGTGCAGCTGGTGCTTCCTCGCTGTTCTTCGTGTACTCCCTTGACT    442

PsANT       ATGCCCGTACCCGATTGGCCAACGACAACAAGTCGGCCAAGGGAGGAGGTGAACGTCAATTCAAAGGTCTCCTCGATGTG    539
SU11-ANT    ATGCTAGGACAAGGCTGGCCAATGACGCAAAGGCATCCAAGGGTGGAGGTGAGAGGCAGTTCAATGGCCTGGTTGATGTC    522

PsANT       TACAAGAAGACCCTGGCTTCCGATGGAATTGGTGGTCTTTACCGTGGTTTCGTCCCCTCAGTCGTTGGTATTGTTGTCTA    619
SU11-ANT    TACCGCAAGACTCTCAAGTCAGATGGTATTGCTGGGCTTTACCGTGGATTCAACATCTCCTGTGTTGGAATCATTGTCTA    602

PsANT       CCGTGGTCTCTACTTCGGAATGTACGATTCGCTCAAACCGGTCGTTCTTACCGGAACCCTCGACGGAAGCTTCTTGGCCT    699
SU11-ANT    CCGTGGTCTGTACTTTGGACTGTATGACTCTCTGAAGCCAGTTCTCCTCACTGGCACTCTCCAGGACAACTTCTTTGCCA    682

PsANT       CTTTCCTTCTCGGATGGGGTGTCACCACCGGTGCTGGTATCGCTTCTTACCCTCTTGACACTATCCGAAGACGTATGATG    779
SU11-ANT    GCTTTGCTCTTGGTTGGTTGATCACCAACGGTGCAGGTCTTGCATCTTACCCCATTGACACCGTCCGCAGAAGGATGATG    762

PsANT       ATGACTTCCGGTGAAAAGGTCCAATACAAGGGAATGGTTGACTGTGGTTCCCAGATTGTCAAGGCCGAAGGTGTTGCTTT    859
SU11-ANT    ATGACCTCTGGAGAGGCTGTCAAGTACAAGAGCTCCTTGGATGCTTTCCAGCAGATCCTGGCGAAGGAGGGTGCCAAGTC    842

PsANT       CTTGTTCAAGGGAGCTGGTGCCAACATCCTCCGTGGTGTTGCCGGCGCTGGTGTCTTGTCGATGTACGACAAGCTCCAAG    939
SU11-ANT    CCTCTTCAAGGGTGCTGGTGCCAACATCCTCCGAGCCATTGCTGGTGCTGGTGTGCTCTCCGGCTATGATCAGCTCCAGA    922

                     HIGS site 2                Stop codon
PsANT       AGCTCCTCTTCGGCAAGGTCTACAAGGGTGGATCAGGTTAAACTTCTCAGCTCGTTCCCCAAAATAATTAACCCACTAGA   1019
SU11-ANT    TCCTCTTCTTCGGCAAGAAGTACGGCTCCGGCGGTGCCTAG.......................................    963

PsANT       CTCTCGTAATGAGAAAATGATTTCAGTCTATCATATGTGTTCAATGTTTTCGCATGATGTTGCCCTTTTAGGCGCTCTTC   1099
SU11-ANT    ................................................................................    963

PsANT       AATTGATTGTTCATCAATTCATGGTCCCTTTTTCACTTGATGCCTGTATGATGAATTTACAGCGGCGACGATTTCCTCTC   1179
SU11-ANT    ................................................................................    963

PsANT       TT                                                                                 1181
SU11-ANT    ..                                                                                  963
```

Supplementary Figure 1 Sequence alignment of PsANT and its homologue in wheat cultivar Su11

Supplementary table 1 The primers used in this study

Name	Sequence (from 5' to 3')*	Applications
PsANT _F	ATGTCGTCTCCTCCAAAGG	Amplification of the ORF of *PsANT*
PsANT _R	TTAACCTGATCCACCCTTGTAG	
PstEF_ qRT_F	ATGCGTATCATGGTGGTGGAGTGA	qRT-PCR of *Pst_EF* transcripts
PstEF_qRT_R	TTCGCCGTCCGTGATATGAGACAA	
PsANT-qRT_F	AGCACCCATTGAGCGTATC	qRT-PCR of *PsANT* transcripts
PsANT-qRT_R	CAAGTGAAAAAGGGACCAT	
PsANT _PUC_F	agatcccgggggggcaatgagatATGTCGTCTCCTCCAAAGG	Construction of PUCPsANT (amplification of the full ORF of *PsANT*)
PsANT _PUC_R	ggcaggtaccTTAACCTGATCCACCCTTGTAG	
PsANT _PUC_110R	ggcaggtaccttaCTCCTTCTTGTAACCGAACAG	Construction of PUCPsANT$_{1-110}$ (amplification of the nucleotides encoding the first 110 amino acids of PsANT)
PsANT _PUC_214R	ggcaggtaccttaGAGGGTTCCGGTAAGAAC	Construction of PUCPsANT$_{1-214}$ (amplification of the nucleotides encoding the first 214 amino acids of PsANT)
PsANT _PUC_111F	agatcccgggggggcaatgagatatgAAGGATGGTTACGGACTCTG	Construction of PUCPsANT$_{111-314}$ (amplification of the nucleotides encoding the 111-314 amino acids of PsANT)
PsANT _PUC_215F	agatcccgggggggcaatgagatatgGACGGAAGCTTCTTGGC	Construction of PUCPsANT$_{215-314}$ (amplification of the nucleotides encoding the 215-314 amino acids of PsANT)
PsANT_pREP3x_F	ccgctcgagATGTCGTCTCCTCCAAAGG	Construction of pREP3x_ PsANT
PsANT_pREP3x_R	cgggatccTTAACCTGATCCACCCTTGTAG	
Bax _ pREP3x_F	ccgctcgagATGGACGGGTCCGGG	Construction of pREP3x_ Bax
Bax _ pREP3x _R	catgtcgacTCAGCCCATCTTCTTCCAGAT	
pREP3x_F	GGAAGAGGAATCCTGGCAT	Universal primers for pREP3x vector
pREP3x _R	CTAAAACTACATCTAACACGAAGGG	
PsANT_pET32a_F	cgcggatccTTGGTTCAGAACCAGGGTGAG	Construction of pET32a_PsANT$_{40-100}$ (amplification of the nucleotides encoding the 40-100 amino acids of PsANT)
PsANT_pET32a_R	gggaagcttTTAGTAATCCTTGAAGGCGAAGTTG	
PsANT-HIGS-F1	atattaattaaAGCACCCATTGAGCGTATC	Construction of BSMV:*PsANT*–as1
PsANT-HIGS-R1	tatgcggccgcCGGATTACGTTGGCAGTGT	
PsANT-HIGS-F2	atattaattaaTCGGCAAGGTCTACAAGG	Construction of BSMV:*PsANT*–as2
PsANT-HIGS-R2	tatgcggccgcCAAGTGAAAAAGGGACCAT	

Note: The underline lowercase letters indicated the restriction sites used.
